# Abstracts/Sommaires/Zusammenfassungen

**DOI:** 10.1155/S1463924678000036

**Published:** 1978


### Abstracts Sommaires Zusammenfassungen

### Page 7

**Automation in clinical chemistry: developments and recent trends,** by F. L. Mitchell

Special conditions and pressures which have existed in clinical chemistry over the last three decades formed a favourable background, and produced the necessary stimulus for the development of laboratory automation systems suitable for applications in many other branches of chemistry. Developments include continuous flow, descrete and centrifugal analysers, and most recently, layer chemistry with its "dry-to-the-touch" advantages. The rapid introduction of computers for laboratory management has allowed unforeseen dramatic improvements in the rate and quality of data handling.

**Automation dans la chimie clinique — développements et tendances récentes.**

Des situations et embarras extraordinaires qui prévalaient dans la chimie clinique les trois décennies précédentes formaient un fond propice pour le développement de systèmes d'automation de laboratoire dans plusieurs autres branches de la chimie. Ces développements comprennent des analysateurs continuels, discrets et centrifuges et, récemment, la chimie couche avec ses avantages de "dry-to-the-touch". L'introduction rapide des ordinateurs pour des buts d'administration du travail permet des améliorations dramatiques imprévues en ce qui concerne la rapidité et la qualité du traitement des données.

**Automation in der klinische Chemie: Entwicklungen und Richtungen**

Aussergewöhnliche Situationen und Bedrängnisse, welche in den letzten drei Jahrzehnten in der klinischen Chemie vorherrschten, bildeten einen günstigen Hintergrund, der den nötigen Anreiz fur die Entwicklung von Laborautomations-Systemen für Applikationen in vielen anderen Zweigen der Chemie hervorbrachte. Diese Entwicklungen umfassen kontinuierliche, diskrete und Zentrifugen-Analysatoren und neuerdings Schicht-Chemie mit ihren Vorteilen des "dry-to-the-touch". Die rasante Einfuhrung von Rechnern zu Zwecken des Labor-Managements ermöglichte unvorhergesehene, dramatische Verbesserungen in Geschwindigkeit und Qualität der Datenverarbeitung.

### Page 10

**Developments in the philosophy of automatic analysis,** by Peter B. Stockwell

Automation in the context of laboratory analysis is defined and the various approaches to automating analysis reviewed. The advantages and disadvantages of the concepts offered by commercial companies, i.e. continuous, discrete, centrifugal and flow injection anlayses are compared. A total systems approach is described and illustrated by reference to examples in the author's laboratory. A recent commercial approach in which the various analytical techniques are divided into a number of discrete steps, is also described. Automation and rationalisation of these steps generates a flexible range of equipment to cope with many varied analytical procedures. The future of automated analytical procedures. The future of automated analytical chemistry requires the proper understanding of the chemistries involved, proper use of microcomputers, the development of any flexible sample pretreatment equipment and the design and development of inert noncorrosible dispensers and pumps.

**Evolution dans la philosophie de l'analyse automatique**

L'automatisation est bien définie dans le contexte de l'analytique de laboratoire et les divers rapprochements a l'automatisation de l'analytique sont discutés. Les avantages et les desavantages des concepts commercialisés comme par exemple l'analyse continuée et discrete, le principe des centrifuges et la téchnique "flow injection" sont comparés. Une solution de système integrale est décrite et illustrée par références aux exemples dans les laboratoires de l'auteur. Une nouvelle solution commerciale, dans laquelle les différentes techniques analytiques sont divisées dans un certain nombre de pas indépendants, est également décrite. L'automatisation et la rationalisation de ces pas ont fait naître un système flexible de modules qui a le but de venir à bout d'un grand nombre de méthodes differentes. L'avenir de la chimie analytique automatisée demande des connaissances relatives a la chimie en question, l'utilisation correcte des microordinateurs, le développement d'appareils universels déstinés à la préparation des échantillons ainsi que le dessein et le développement d'appareils de dosage et des pompes inertes et noncorrosifs à la fois.

**Entwicklungen in der Philosophie der Automatische Analyse.**

Automation wird im Zusammenhang mit Laboranalytik definiert und die verschiedenen Ansätze zur Automation der Analytik besprochen. Die Vor- und Nachteile der kommerziellen Konzepte wie kontinuierliche und diskrete Analysen, Zentrifugenprinzip und "flow injection" Technik werden verglichen. Eine integrale Systemlösung wird beschrieben und illustriert durch Hinweis auf Beispiele in den Laboratorien des Autors. Eine neue kommerzielle Lösung, bei der die verschiedenen Analysentechniken in eine Reihe von Einzelschritten aufgelöst werden, ist ebenfalls beschrieben. Automation und Rationalisierung dieser Schritte lassen ein flexibles System von Geräten entstehen, um damit die verschiedensten Analysenmethoden bewaltigen zu konnen. Die Zukunft der automatisierten analytischen Chemie verlangt einschlägige Kenntnisse der betroffenen Chemie, richtigen Gebrauch der Mikrocomputer, die Entwicklung von vielseitigen Probenvorbereitungsgeräten und Entwurf und Entwicklung von inerten, korrosionsfesten Dosiergeräten und Pumpen.

### Page 14

**High-speed automatic dispenser/diluter or dual pipetter/mixer,** by David L. Krottinger, Michael S. McCraken and Howard V. Malmstadt.

A versatile automated unit that can be used as either a dual pipetter/mixer or dispenser/diluter has been constructed and evaluated. The unit is portable and has a refill-delivery cycle of about 2 seconds, and can be operated by microcomputer control or by manual push-button operation. Discrete quantities of solution over a wide range of volumes (for example 0.05 - 1 ml per syringe) can be delivered and mixed with excellent precision, 0.1% RSD or better.

**Appareil automatique rapide pour l'usage comme double pipette avec mélangeur, soit comme dispenseur-diluteur**

Un appareil universel et automatique qui peut être utilisé soit comme double pipette avec mélangeur, soit comme dispenseur-diluteur a été construit et techniquement étudié. L'appareil est portable, le cycle de remplissage et dosage est de 2 secondes. Il peut être mis en oeuvre par un contrôle externe avec microordinateur ou manuellement par boutonpressoir. Les quanitités de solution delivrées sont comprises dans une large gamme de volume (par exemple de 0,05 à 1 ml par seringue) et mélangées avec une excellente précision (0,1% CV ou mieux).

**Ein schnelles automatisches Dosier/Verdünnungsgerät oder zweifaches Pipettier/Mischgerät.**

Ein vielseitiges, automatisches Gerät, das entweder als zweifaches Pipettier-/Mischgerät oder Dosier-/Verdünnungsgerät verwendet werden kann, wurde gebaut und evaluiert. Das Gerät ist tragbar und hat eine Zykluszeit für Füllen-Dosieren von etwa zwei Sekunden. Es kann entweder durch eine Mikro-Computersteuerung oder manuell durch Drucktasten bedient werden. Über einen weiten Bereich von Volumina (z.B. 0,05 - 1 ml pro Dosierspritze) können mit einer ausgezeichneten Präzision von 0,1% relative Standard-Abweichung oder sogar besser wiederholbare Quantitäten ausgestossen und vermischt werden.

### Page 22

**An automated sampler for high temperature headspace gas chromatography,** by J. B. Pausch, R. E. Arner and R. P. Lattimer.

An automated headspace analyzer has been constructed for the purpose of analysing a

**Un échantillonneur automatique pour l'analyse par chromatographie en phase gazeuse des "surnageants".**

Un analyseur automatique des surnageants a été construit dans le but d'analyser une série

**Ein automatischer Probenzieher für Dampfraum-Gaschromatographie.**

Für die Zwecke der unbeaufsichtigten Serieanalyse von Proben mit Dampfraum-Gas-

### Page 22 continued series of samples by headspace gas chromatography without operator intervention. This technique is particularly useful for determining trace amounts of residual chemicals in polymers and latexes, and chemicals released from solid adsorbents. The features of this unit include a high temperature oven and independent digital control of the sampling system. This latter feature enables the equipment to stand alone and thus, it is adaptable to any existing gas chromatograph.

d'échantillons par chromatographie en phase gazeuse sans l'intervention d'un opérateur. Cette technique est particulièrement utile pour l'analyse de traces de résidus chimiques dans les polymères et les latex, aussi que les produits chimiques dégagés d'absorbants solides. Parmi les caractéristiques de cette unité on trouve un four à haute température et un contrôleur digital indépendant du système échantillonneur. Cette derniere qualité permet une utilisation indépendante et l'appareil s'adapte à tout appareil de chromatographie en phase gazeuse existant.

chromatographie wurde ein automatischer Dampfraum-Analysator konstruiert. Diese Technik ist besonders fur die Bestimmung von Spurenmengen von Chemikalien in Polymeren und Latexen sowie von Chemikalien, die Festkörper-Absorbern entstammen, nützlich. Die Merkmale dieser Einheit umfassen einen Hochtemperaturofen und eine unabhängige digitale Steuerung des Probeneinlasssystems. Diese letzte Eigenschaft macht dieses Gerät eigenständig und es kann auf diese Art und Weise an jeden beliebigen Gaschromatographen angepasst werden.

### Page 28

**Automated solid-liquid extraction system,** by H. Bartels, R. D. Werder, W. Schurmann and R. W. Arndt

An automatic unit is presented for solid-liquid extraction as a step of sample preparation in chemical analysis. The unit consists of a mill and a centrifuge for separation of the phases. The controls allow automatic individual treatment without supervision of a wide variety of samples. The quality of the results exceeds those obtained by manual methods.

**L'extraction automatique solide-liquide**

Une unité automatique est présentée pour l'extraction solide-liquide qui a pour but de préparer des échantillons dans des analyses chimiques. L'unité consiste d'un broyeur et d'une centrifugeuse pour la séparation des phases. La commande permet un traitement automatique et individuel sans surveillance d'une large diversité d'échantillons. La qualité des résultats est superiéure à ceux obtenus par des méthodes manuelles.

**Automatische fest-flüssig Extraktion**

Ein automatisches Gerät für die fest-flussig Extraktion als Probenvorbereitungsschritt in der chemischen Analyse wird vorgestellt. Die Einheit besteht aus einer Mühle und einer Zentrifuge fur die Trennung der Phasen. Die Steuerung erlaubt eine automatische, individuelle Behandlung ohne Überwachung eines breiten Spektrums von Proben. Die Qualität der Resultate ist besser als diejenigen, die mit manuellen Methoden errecht werden.

### Page 32

**Practical and organisational problems in testing clinical laboratory instruments,** by L. B. Roberts.

This paper examines some of the non technical factors which must be taken into account when performing evaluations on clinical laboratory instruments. Eighteen different components associated with evaluations are considered, examples being publication of report, relationships with manufacturers, costs in evaluation, insurance and staffing, etc. The paper is intended to act as a guide to staff in laboratories which are intending to carry out evaluatory work.

**Problèmes de practique et d'organisation dans l'épreuve d'instruments de la laboratoire clinique.**

Dans cet étude l'auteur examine quelques facteurs non-techniques dont on doit se rendre compte en formant des évaluations des instruments de la laboratoire clinique. Il considère dix-hit components divers associés avec des évaluations, par example la publication des rapports, les rélations avec les fabricants, les frais d'évaluation, les assurances,, tous concernant le personnel. Cet étude a pour le but de rendre assistance au personnel des laboratoires qui ont l'intention d'évaluer les dites instruments.

**Praktische und organisatonische Problemen im Proben der Klinischen Laborsinstrumenten**

Diese Schrift prüft einige der nicht technischen Faktoren, die in Betracht ziehen müssten, wenn Wertbestimmungen mit klinischen Laborsinstrumenten gemacht werden. Achtzehn verschiedene Komponenten, die mit Wertbestimmungen verbunden sind, werden betrachtet, zum Beispiel Berichtpublikation, Herstellerverwandtschaften, die Kosten fur die Wertbestimmung, Versicherung und Angestellten u.s.w. Die Schrift ist gemeint als eine Führung für Angestellten in Laboratorien wo Wertbestimmungarbeit gemacht wird.

### Page 36

**The Vickers SP120 Analyser: an instrument evaluation,** by B. P. Ager, R. Gentle, E. W. Ingarfill and A. L. Tárnoky

The Vickers SP 120 is an attachment designed to modernise the Technicon SMA series of analysers. We have tested an SP 120 - SMA 12/60 complex. It operates at the faster rate of 120 specimens/h, and reduces the size of samples (including those of standard and control sera) and the volume of reagents. These points and its less labour intensive operation give significant saving in costs and working time, without overall loss in the quality of results.

**L'Analyseur Vickers SP120: une étude technique.**

Le Vickers SP120 est une accessoire complémentaire destinée à moderniser la serie Technicon SMA. Nous avons testé le SP120 avec le SMA 12/60. Ce système fonctionne à un système accéléré de 120 specimens par heure et réduit la grandeur de l'échantillon (celle des sérums standard et de contrôle) et le volume de réactif. Ces caractéristiques, ajoutées à l'opération manuelle reduite, permettent des economies sensibles du coût et de la durée du travail sans affecter la qualité des résultats.

**Der Vickers SP 120 Analysator: eine Instrumentabschatzung.**

Der Vickers SP120 ist ein Zusatzgerät, das die Technicon Analysatoren der Serie SMA modernisieren soll. Wir führten Testversuche an einem solchen SP120-SMA 12/60 Komplex durch. Er arbeitet bei der höheren Durchgangsgeschwindigkeit von 120 Proben pro Stunde und reduziert die Probenvolumina (einschliesslich derjenigen der Standard- und Kontrollseren) als auch die Volumina der Reagenzien. Diese Punkte und die weniger arbeitsintensive Benützung ermoglichen signifikante Einsparungen an Kosten und Arbeitszeit ohne Einbusse in der Qualität der Resultate.

### Page 40

**Assessment of the Chemetrics analyser,** by Robyn White, N. Potezny, T. D. Geary, D. Elston and B. Fuller.

The Chemetrics analyser is a versatile, simple to operate instrument which would require very little maintenance. It is preprogrammed to perform a wide variety of tests but with the added feature of allowing the operator to enter the laboratory's own methods through the keyboard. The instrument performed creditably during the evaluation producing results of a high standard with respect to both precision and accuracy.

**L'evaluation de l'analyseur Chemetrics.**

L'analyseur Chemetrics est un instrument universel et facile à manier qui nécessite peu d'entretien. Il est programmé d'une facon qu'un grand spectre de méthodes peut etre exécuté. La possibilité additionnelle est donne a l'operateur d'employer, moyennant un clavier, les méthodes existantes dans son propre laboratoire. Pendant l'évaluation, l'instrument a donné des resultats remarquables d'une haute précision.

**Die Einschatzung des Chemetrics Analysators.**

Der Chemetrics Analysator ist ein vielseitiges, leicht zu bedienendes Instrument, das wenig Unterhalt erfordert. Er ist so programmiert, dass ein grosses Spektrum von Methoden bearbeitet werden kann, wobei zusätzlich die Möglichkeit gegeben ist, via Tastatur die eigenen im Laboratorium vorhandenen Methoden durch die Bedienungsperson zur Anwendung zu bringen. Während der Evaluation erbrachte das Instrument zuverlässig Resultate von hoher Präzision und Genauig keit.

